# Awake Craniotomy for Resection of Cerebral Arteriovenous Malformation: Initial Experience From a Low- and Middle-Income Country

**DOI:** 10.7759/cureus.17596

**Published:** 2021-08-31

**Authors:** Saqib Kamran Bakhshi, Mishaal Ather, Quratulain Tariq, Saad Bin Anis, Syed Ather Enam

**Affiliations:** 1 Neurosurgery, Aga Khan University Hospital, Karachi, PAK; 2 Neurosurgery, Shaukat Khanum Memorial Cancer Hospital and Research Centre, Karachi, PAK

**Keywords:** awake craniotomy, arteriovenous malformation, spetzler-martin grade, digital subtraction angiography, global neurosurgery

## Abstract

Global health has shown progress over the years; however, neurosurgical care has not followed the same trajectory due to it being presumably resource intensive. Awake craniotomy (AC) is a neurosurgical technique that can improve neurological outcomes, can potentially reduce costs and hospital stay, and can be easily employed in low- and middle-income countries (LMICs). It has proven to be beneficial in surgical resection of tumors located in the critical areas of the brain, but there is limited literature to support AC for resection of arteriovenous malformations (AVM). We present four cases of AVM that were successfully treated surgically under awake settings in a developing country. Two of the AVMs were Spetzler-Martin grade (SMG) 3, one was SMG 4, and one was SMG 1.

All the patients underwent successful excision of AVMs, and the postoperative digital subtraction angiography (DSA) was negative for any residual. They had a total hospital stay of three to five days with a mean postoperative stay of two days. Only one patient showed transient conductive dysphasia, which resolved on subsequent follow-ups, and none of the patients developed any long-term neurological deficit.

There are limited data from LMICs regarding the benefits of using AC for AVMs. However, our cases show that this technique can be applied for AVM resection, particularly in eloquent areas of the brain (parts of the cerebral cortex that control vision, language, sensory, and motor functions), to minimize potential neurological deficits. Even though it requires careful selection of cases, and needs a higher level of microsurgical and neuro-anesthesia expertise, it can lead to better postoperative outcomes, lesser morbidity, and a shorter hospital stay, contributing to low resource utilization, making it feasible in a resource-limited setting.

## Introduction

Global health has shown remarkable progress in improving health worldwide, however, the improvement has not been uniform [1]. While most of the progress has been seen in reducing the burden of infectious diseases, improvement in surgical care has been suboptimal, with countries having a gross national income (GNI) per capita of less than $12,375, commonly referred to as low- and middle-income countries (LMICs) by the World Bank, being affected most drastically. Due to high perceived resource utilization and associated costs, it is considered a low priority in LMICs [1].

Neurosurgical care is further neglected worldwide and is considered as an entity only for the privileged. This is despite the fact that neurological diseases are the leading cause of disability-adjusted life years (DALYs), and the second leading cause of death globally [2]. Furthermore, without appropriate efforts, it is estimated that the economic burden of neurological disorders would sum up to be a total of US$12.3 trillion between 2015 and 2030 [1]. In addition, LMICs are faced with barriers that make implementing or developing neurosurgical care difficult. These include lack of resources, infrastructure, finances, sophisticated equipment, technology, all of which are required for timely diagnosis and appropriate management [3].

Awake craniotomy (AC) is a neurosurgical technique that has the potential to be useful in resource-limited settings. It was historically introduced to treat seizures but is now widely used for various intracranial pathologies, including brain tumors and arteriovenous malformations (AVMs) [4]. It involves applying scalp blocks for pain relief so that the patient is kept awake during the procedure for monitoring neurological functions. This enables accurate cortical mapping that guides neurosurgeons to make appropriate intraoperative decisions, leading to, reduced risk of complications and improved outcomes [5]. The most important indication for performing AC is to avoid new-onset neurological deficits. Additionally, other benefits include avoidance of general anesthesia (GA), reduced operation time, decreased requirement of extensive monitoring devices, less need for intensive care unit postoperatively, and shortened recovery time [4]. These advantages subsequently result in reduced healthcare expenditure, making AC a feasible modality in countries with limited resources [6].

While there are studies from high-income countries that highlight the benefits of AC, there are inadequate data from LMICs, the majority of which is regarding brain tumors. In this study, we have reported our experience of employing AC in treating four cases of AVMs in an LMIC. The ill-defined natural progression of unruptured AVMs and the risk of morbidity and mortality associated with their possible location in eloquent areas of the brain have posed challenges in choosing the most appropriate treatment option [7]. Eloquent areas are those parts of the cerebral cortex that directly control major functions of the human body, including language, vision, sensory, and motor functions. AC has shown to be successful in facilitating careful excision and preventing neurovascular compromise in the adjacent areas of the brain, thus improving outcomes [8].

## Case presentation

It is a prospective series of four cases performed at our center. Two of the patients had AVMs of Spetzler-Martin grade (SMG) 3, one had an AVM of SMG 4, located in the eloquent area, and one had an AVM of SMG 1. All patients underwent neuro-navigation guided AC with intraoperative neurological monitoring for excision of their AVMs at a tertiary care center (Aga Khan University Hospital, Karachi) in an LMIC.

Case 1

**Figure 1:**
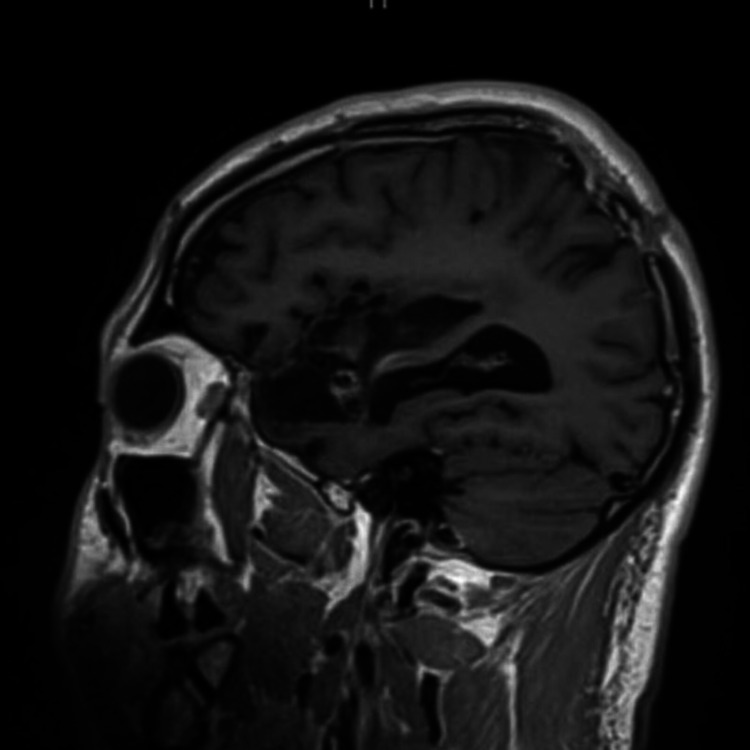
Right frontal arteriovenous malformation (case 1) T1 weighted sagittal section of magnetic resonance imaging, demonstrating arteriovenous malformation in the right frontal area, superior and posterior to the encephalomalacia.

A 32-year-old male patient presented with a history of subarachnoid hemorrhage due to a ruptured right middle cerebral artery (MCA) aneurysm a year back. He had undergone coiling of the aneurysm, decompressive craniectomy, and subsequent cranioplasty, at another hospital setting. On a follow-up scan one year later (Figure 1) he was found to have a right frontal AVM of SMG 3 (size of 3 cm, superficial venous drainage, and eloquent location), which had not been picked up on earlier scans, and presented to us for further treatment. He had no focal neurological deficits but complained of frequent headaches.

**Figure 2:**
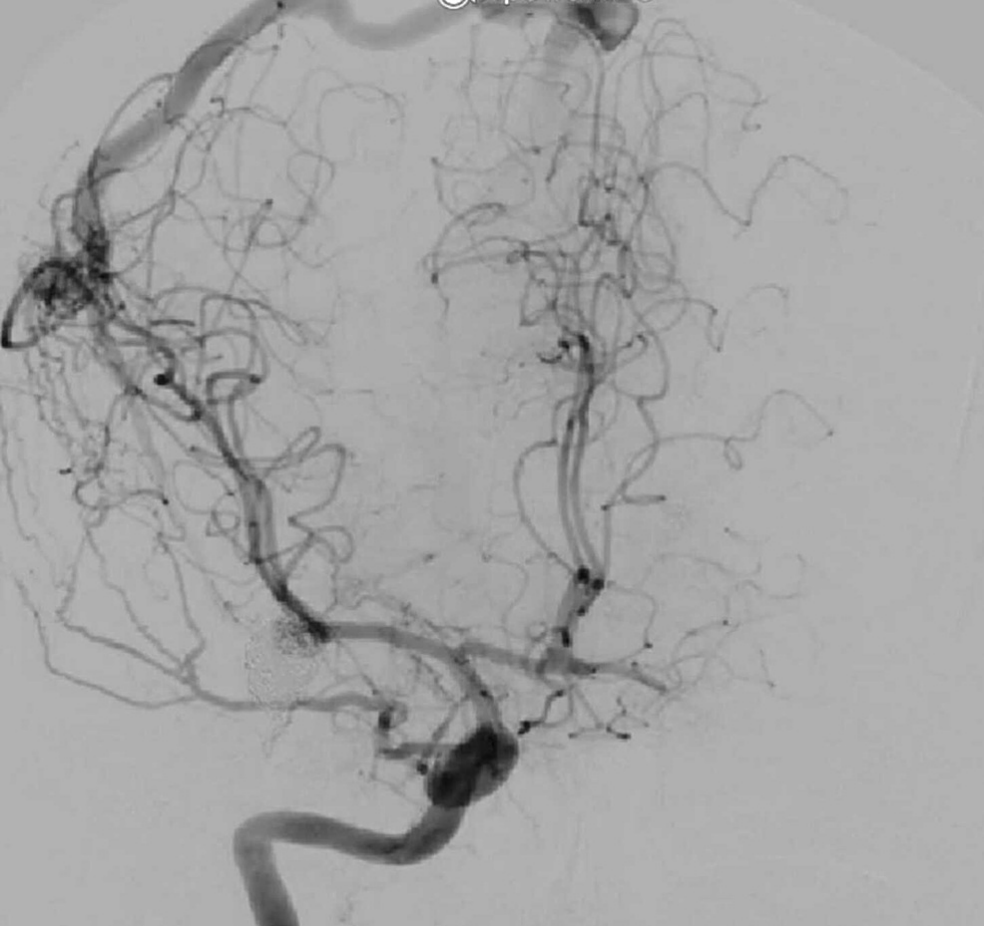
Right frontal arteriovenous malformation (case 1) Pre-operative digital subtraction angiogram of right internal carotid artery showing the arteriovenous malformation as well as the coiled middle cerebral artery aneurysm.

The patient underwent excision of the AVM via neuro-navigation-guided AC. Intraoperative findings were a right frontal AVM, with the size of the nidus being 3 × 3 cm^2^, which was supplied by a branch of the MCA and was draining into the superficial venous system. A temporary clip was applied to one major branch supplying the nidus to assess the development of intraoperative neurological deficits (Figure 2).

**Figure 3:**
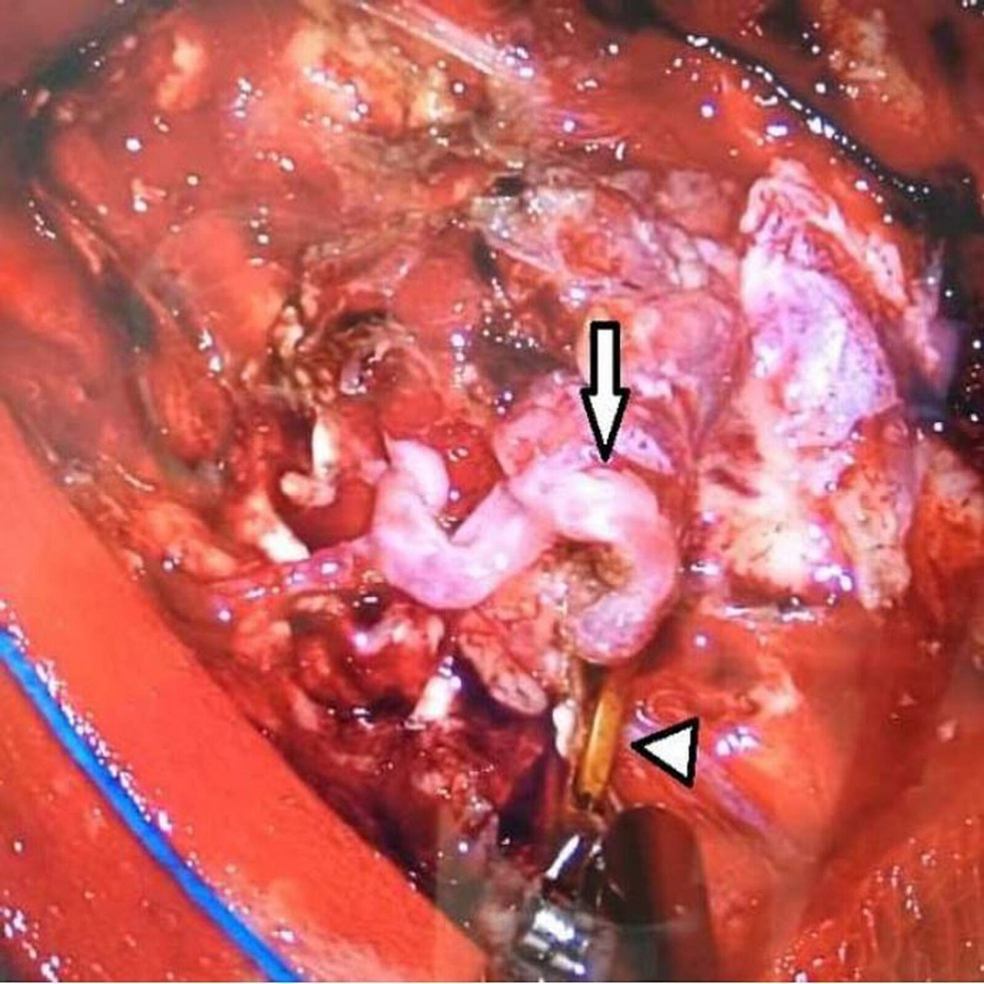
Temporary clip application on the feeding artery (case 1) Intra-operative view showing temporary clip (arrowhead) on the artery feeding the nidus (arrow) of arteriovenous malformation, to evaluate for the development of any neurological deficit.

Motor control on the left side in both extremities, as well as speech and comprehension, was monitored for five minutes. When it was found that the patient did not develop any deficit due to temporary clipping, the clip was removed, and the artery was carefully coagulated. The surgery was completed without any complications, and postoperative digital subtraction angiogram (DSA; Figure 3) was negative for any residual AVM. The patient was discharged without any neurological deficits within four days of admission to the general ward.

Case 2

**Figure 4:**
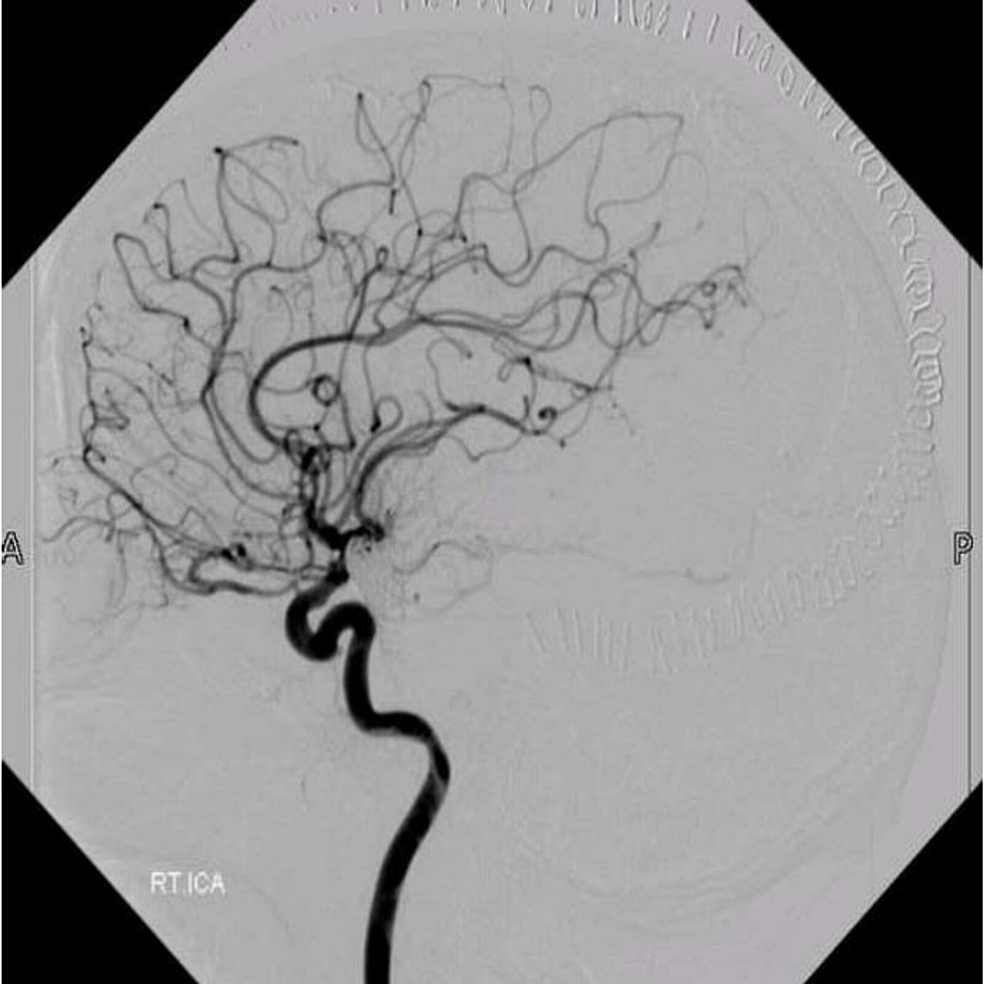
Post-operative digital subtraction angiogram (case 1) Post-operative digital subtraction angiogram of the right internal carotid artery, showing no residual arteriovenous malformation.

A 20-year-old female patient presented with complaints of intermittent left-sided headaches for two years, and blackouts associated with transient dysphasia for three months. She had also reported two episodes of generalized tonic-clonic seizures within the preceding two weeks, that lasted for a few minutes. The patient was otherwise well and was on Methylphenidate for six months. The patient underwent a DSA and was diagnosed with left parieto-temporal AVM of SMG 4; size more than 3 cm, presence in the eloquent cortex, and venous drainage to the vein of Galen apart from superficial drainage (Figure 4). The AVM was found to be supplied by the left MCA and left posterior cerebral artery (PCA). Pre-operative MR images were not available for this case series.

**Figure 5:**
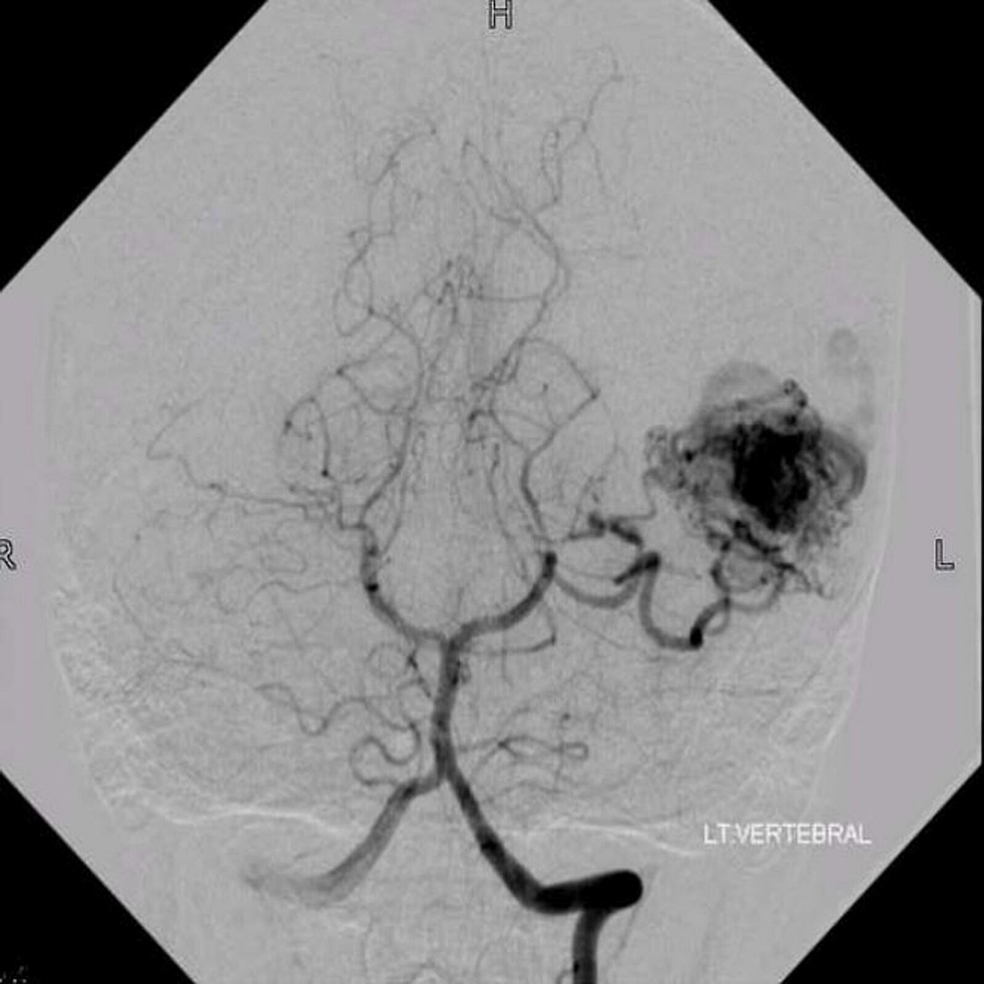
Pre-operative digital subtraction angiogram (case 2) Pre-operative angiogram of the left vertebral artery, showing arteriovenous malformation in the left temporal region of a nidus measuring approximately 36 × 14 mm^2^ primarily fed by posterior cerebral artery.

She was started on anti-seizure medications and was advised surgical excision. The patient underwent angioembolization of the AVM prior to surgery. Approximately 80-90% of the AVM, including nidus, was embolized with ONYX, and the residual AVM was filled by the left MCA feeder and minor feeders of left PCA, as seen on the DSA (Figure 5).

**Figure 6:**
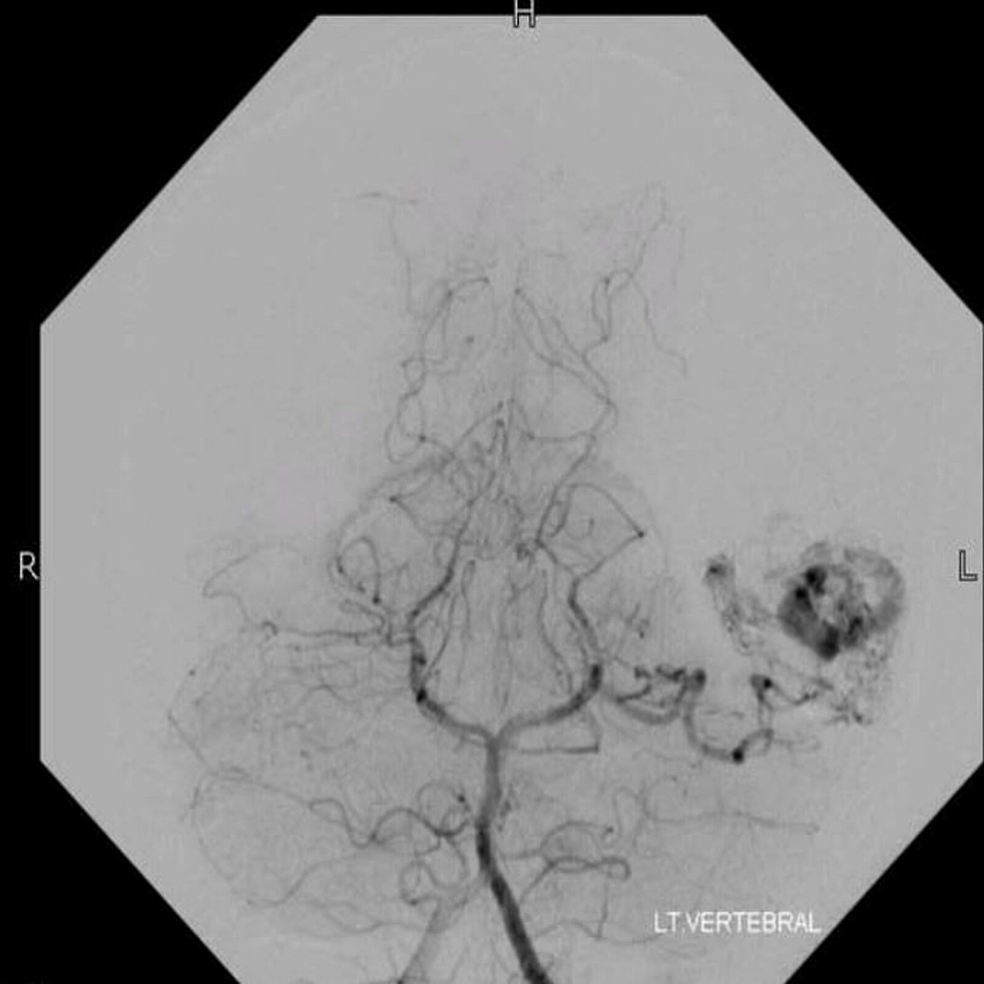
Post-embolization digital subtraction angiogram (case 2) Cerebral angiography after embolization showed approximately 80-90% obliteration of malformation.

The patient subsequently underwent neuro-navigation guided AC and excision of AVM. Cortical mapping was done on the brain surface to identify the speech area, and corticectomy for AVM resection was performed at an area that was negative for speech function. Intraoperative findings were abnormal tortuous, dilated, malformed tuft of vessels in the left temporal region, along with small feeders on the anterior and posterior parts, and dilated reddish-blue vessels engorged with ONYX. A temporary clip was placed on the branches of MCA and no deficit was seen clinically. Postoperatively, the patient remained stable initially, but later developed conductive dysphasia and memory impairment, six hours after the procedure. A plain CT scan of the brain showed cerebral edema without any significant hemorrhage. She was then started on Dexamethasone that resolved her symptoms in 36 hours. DSA on the second postoperative day showed no residual AVM (Figure 6). The total length of hospital stay was five days, and she was discharged with no neurological impairment.

Case 3

A 32-year-old female patient presented with complaints of throbbing headache in the right parietal region for the preceding three months. She was diagnosed with right posterior parietal AVM of SMG 3 based on magnetic resonance imaging (MRI) of the brain and CT angiogram; size more than 3 cm, presence in the eloquent region, and superficial venous drainage.

**Figure 7:**
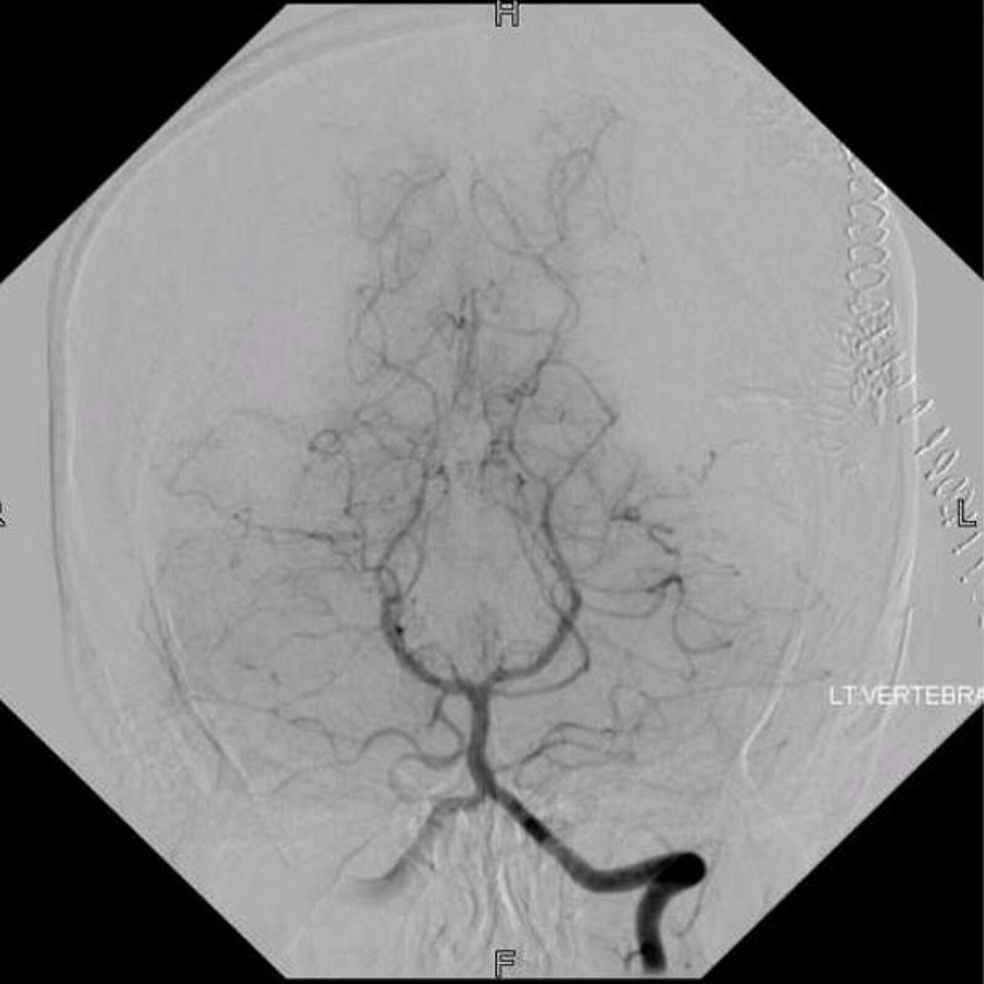
Post-operative digital subtraction angiogram (case 2) Post-operative angiography revealing complete excision of previously noted arteriovenous malformation in the left temporal region.

The patient initially underwent DSA (Figure 7), which confirmed an AVM in the right posterior parietal lobe, predominantly supplied by right MCA and draining into superficial sagittal sinus.

**Figure 8:**
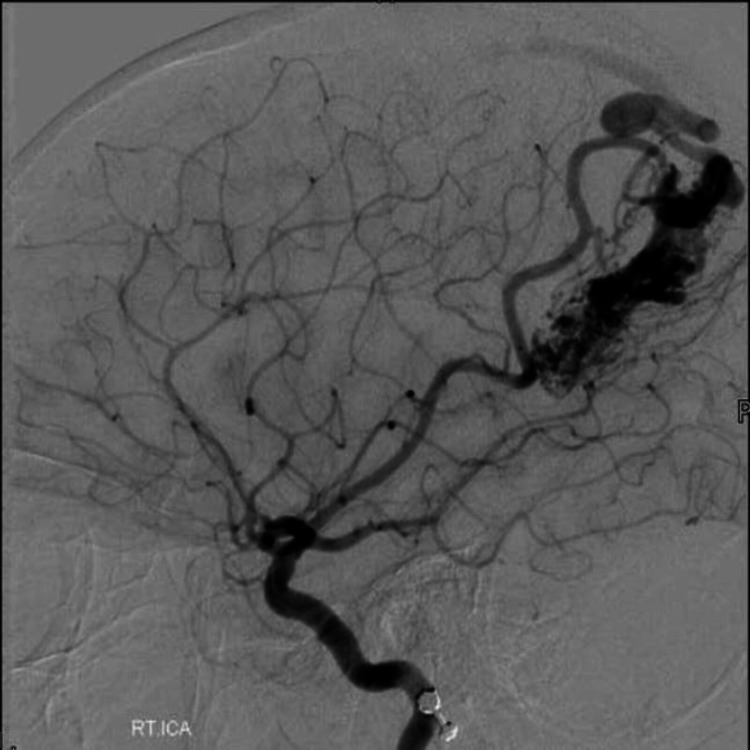
Pre-operative digital subtraction angiogram (case 3) Pre-operative digital subtraction angiography of the right internal carotid artery, showing arteriovenous malformation in the right posterior parietal lobe. It was primarily being fed by the middle cerebral artery.

Neuro-navigation guided right parietal AC and excision of AVM was planned. The intraoperative findings were the same as that of the DSA. The feeding vessel was temporarily clipped during surgery to look for any neurological deficit, before being coagulated. The entire nidus was completely resected as confirmed by a postoperative DSA (Figure 8).

The patient remained well postoperatively, except for facial pain in the distribution of the ophthalmic branch of the trigeminal nerve, likely due to head clamp placement, which improved over time. The total length of hospital stay was four days, and the patient was discharged without any neurological deficits.

Case 4

**Figure 9:**
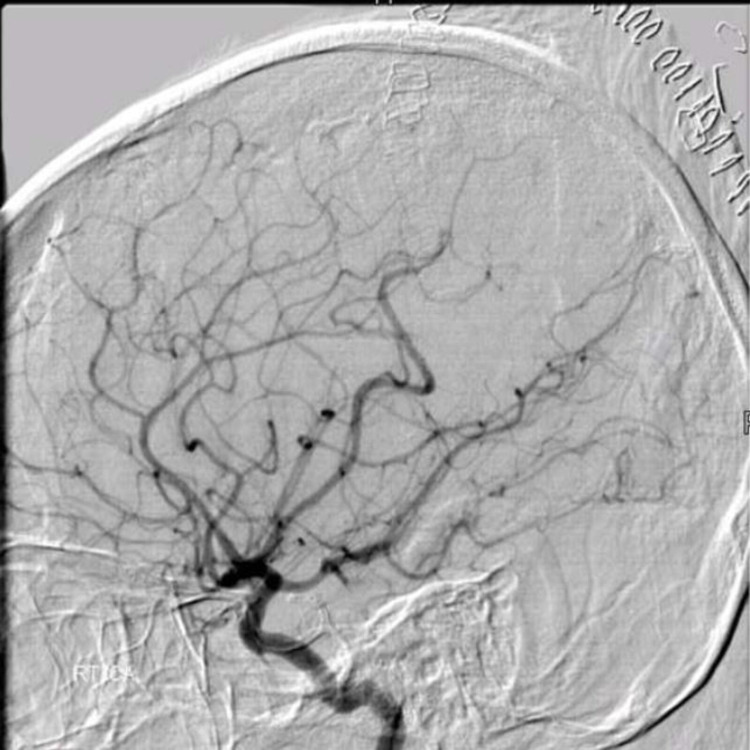
Post-operative digital subtraction angiogram (case 3) No residual malformation was seen in the postoperative digital subtraction angiogram.

A 27-year-old female patient, presented with complaints of intermittent headaches of moderate intensity for two years. She underwent MRI brain with contrast (Figure 9) and CTA and was diagnosed with pial AVM in the right frontal lobe of SMG 1; size less than 3 cm, non-eloquent location, and superficial venous drainage. Since it was a superficial and very small AVM, an angiogram was not done pre-operatively.

**Figure 10:**
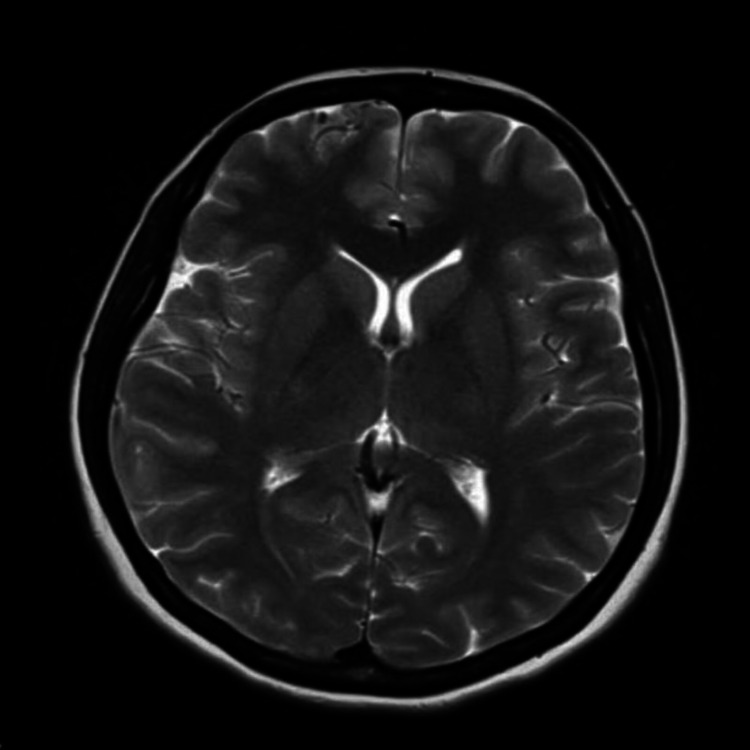
MRI showing right frontal arteriovenous malformation (case 4) T2-weighted axial section of MRI, showing evidence of a small bunch of vessels with a prominent dilated vein at the right frontal lobe anteriorly. Appearances are suggestive of small pial arteriovenous malformation.

**Figure 11:**
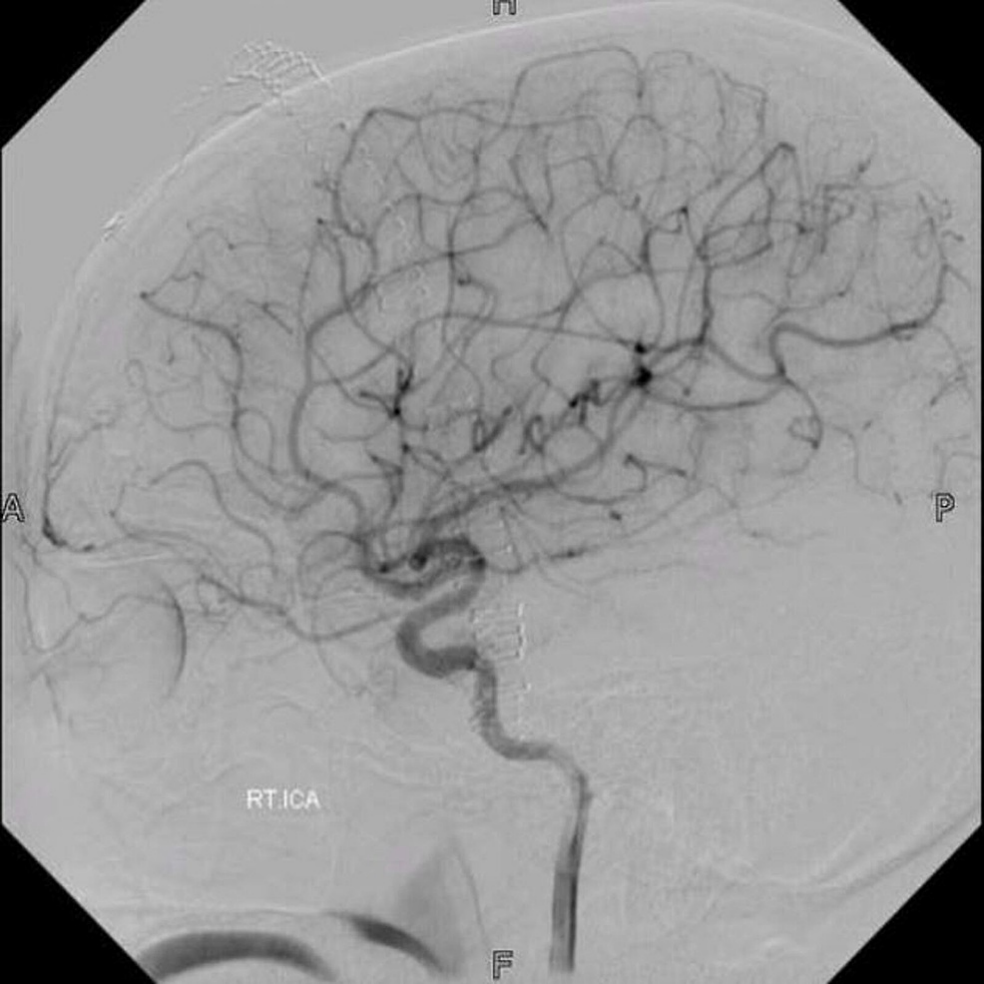
Postoperative digital subtraction angiogram (case 4) Post-operative cerebral angiogram of the right internal cerebral artery in which no residual malformation is seen in the right frontal region.

She underwent neuro-navigation-guided right frontal AC and excision of AVM. Intraoperative findings were a tuft of tortuous, dilated blood vessels in the right frontal region, showing reddish-blue blood representing the AV shunts, with venous drainage towards the dura mater. No intraoperative temporary clip was needed. Postoperatively, the patient did well with no neurological deficits except weakness of the supra-orbital nerve which affected her frowning. She underwent DSA postoperatively, and no residual malformation was seen (Figures 10 and 11). Her total length of hospital stay was three days.

## Discussion

AC with brain mapping allows for real-time localization of eloquent areas of the brain, which enables safe surgical excision, and decreased risk of postoperative neurological deficit [9]. This is achieved by joint efforts of the neurosurgery and the anesthesia team, to keep the patient awake during the procedure, and monitor responses and development of neurological deficits during the course of surgery. Not only does this reduce the surgical time, but it also prevents patients from having the risk of developing complications of GA, and contributes to a shorter recovery time and reduced hospital stay. While we could not find any data comparing hospital stay between AVM resection under scalp block vs GA, a study by Ackerman et al. on brain tumor excision reported the average length of stay of two days after AC as compared to 8.3 days for craniotomy under GA [10].

It is due to these benefits that make AC a resource-conservative treatment modality. There have been studies in LMICs that highlight the feasibility and successes of AC in resource-limited countries [11]. In a study by Howe et al., AC was taught and sustainably implemented in six centers located in four countries; Indonesia, Ghana, Nigeria, and China. Thirty-eight AC were performed for intracranial tumors. There was no mortality; the most common postoperative complication was a seizure, and only one patient developed a permanent neurological deficit. There were reduced central line insertions, and 64% of the patients had a hospital stay of less than ten days. A senior staff educator was present during some of the surgeries but subsequently, the countries were able to conduct the surgeries independently, reflecting the sustainability of AC [12]. Another study from Brazil stressed that a lack of specialized equipment and personnel such as neurophysiologists, neurologists, neuropsychiatrists, or speech therapists does not prevent the success of cortical mapping during the procedure [13].

While there have been some studies in the developed countries that highlight the benefits of AC in treating AVMs, there are no data from resource-limited countries, as most of them have been done on patients with brain tumors [11-13]. In our study, we describe the success of AC in excising AVMs in an LMIC. AVMs, though a rare congenital anomaly of the brain, are of great interest, as the natural course of unruptured AVMs is quite variable, making treatment options controversial. The various treatment modalities available for the management of AVMs include microsurgical excision and stereotactic radiosurgery, with angioembolization an important adjunct to treatment. The outcomes of surgery largely depend on the size of the AVM, arterial feeders, deep venous drainage, and size and compactness of the nidus [7]. The risk associated with surgery can be predicted using various grading systems, however, the most commonly used system is the SMG system [8]. Risk is higher in AVMs located in the eloquent area of the brain in patients with SMG 3 to 5, however, location does not change the risk in patients with SMG 1 and 2 [7].

In lieu of the risks of surgery, AC can be beneficial in reducing intraoperative and postoperative complications, particularly for those located in eloquent areas. In a case series by Burchiel et al., eight patients, with AVMs located in the sensorimotor and language cortex, were operated on, and only one patient developed neurological deficit postoperatively, which also resolved on subsequent follow-ups [14]. Similar findings were published in a case series by Gamble et al. Four patients of all the AVM patients studied in their series, had AVM on the left side in the language area and underwent cortical mapping and AC. Complete resection was seen in all four of them with no long-term neurological deficits [15]. Another retrospective study showed that patients with AVMs located in the eloquent area had preserved neurological functions when operated using the awake anesthesia technique [16].

In our study, the patients with AVM of SMG 3 and 4 were at a high risk of neurological deficits post-surgery, which made them suitable candidates for AC. However, even the patient with the AVM of SMG 1 was performed under awake settings despite the location not posing any risk of neurological deficits. This was because AC allows easy access to superficial AVMs, making resection easy and allowing patients to avoid complications associated with GA, and is, therefore, a good method of treatment even if the AVM is located in a non-eloquent area. Additionally, the AVMs that are not present in the eloquent area can have branches supplying the eloquent areas, which can put patients at risk of developing neurological deficits, and cortical mapping may be performed in such cases. In our study, AC were successfully performed in all the patients and all AVMs were resected without any residual AVM as confirmed by postoperative DSA. Only one patient showed transient conductive dysphasia. None of the patients had permanent neurological deficits secondary to excision of the AVM nidus.

In addition, AVMs that have bled are easier to resect because the gliotic tissue provides a plane for easy resection [17]. With the exception of one case, who had a history of subarachnoid hemorrhage, secondary to aneurysm, the patients in our cases had unruptured AVMs, which made them challenging to operate. However, approaching resection via AC was an attempt to prevent postoperative neurological deficits, which was successfully achieved as none of the patients developed any long-term morbidities. Temporary clipping of the feeding artery is another way for neurosurgeons to assess for any neurological deficits, and this was done for two of the patients in this series.

The patients who present with seizures, such as the female patient with a grade 4 AVM among our cases, need to be dealt with consideration and care, and surgery needs to be done at the gliotic region immediately surrounding the nidus. In addition, seizures are one of the serious complications that can occur during surgery and can be associated with electrical stimulation. It carries the possibility of the AC being converted into GA, which makes the role of the anesthesiologist team critical in taking adequate measures timely [18]. In contrast to some of the studies in LMIC, none of the patients in our study developed seizures intraoperatively [3]. The importance of neuro-anesthesia expertise cannot be undermined, as there are complications reported in the literature such as obstructive apnea, nausea, vomiting, seizures, and loss of consciousness intraoperatively. The patient’s anxiety and agitation during the operation also need to be addressed effectively with the appropriate management of analgesia and sedation [19]. Furthermore, AC requires a higher level of expertise, particularly psychomotor control of the surgeon, and equipment to be able to carry out the procedure in a lesser time.

With respect to socio-economic benefits of AC, we did not investigate the cost of surgery as we did not have a comparative group (AVM resection under GA) to look for any difference in costs, but we looked up the length of stay which is an indicator of resource utilization. AC, though a very useful modality, does have various challenges and hurdles, particularly in developing countries. These include lack of neuro-anesthesia expertise, and low literacy rate, among others [20]. However, strengthening neurosurgical capacities in LMICs is imperative to ensure equitable care is provided to patients.

Based on the cases reported and their outcomes, our recommendations are that patients with superficial AVMs located in or adjacent to eloquent areas, are the best candidates for AC, and should be treated via this approach, as it would benefit the patient by reducing the risk of developing neurological deficits postoperatively, and reduce the overall cost of patient care. Larger AVMs, however, are still difficult to approach using the awake technique as their resection is time-consuming, and therefore, could be continued to be treated under GA. However, more studies are needed to evaluate this and to come up with more specific recommendations regarding indications of AC for AVM resection. Additionally, efforts and collaborations of experienced neurosurgeons and anesthesiologists are needed to implement AC in countries of limited resources.

The strength of this case series is that the procedures were done by a single surgeon, which reduces inter-procedural variability. However, the number of patients were less, and further studies with a larger sample size need to be conducted for generalizable results.

## Conclusions

AC is associated with better postoperative outcomes when used to treat AVMs located in the eloquent areas. It can also be employed for superficial AVMs, and those located in non-eloquent regions, to avoid GA and its associated complications. The hospital stay and extent of monitoring required after AC are significantly less as compared to AVM resection under GA, which results in lower cost.

Careful selection of cases is needed to ensure maximum benefit of AC. Furthermore, it requires a higher level of sophistication and expertise, which can be established and safely achieved by surgeons with experience.
